# Sialic acid-engineered mesoporous polydopamine dual loaded with ferritin gene and SPIO for achieving endogenous and exogenous synergistic T2-weighted magnetic resonance imaging of HCC

**DOI:** 10.1186/s12951-021-00821-8

**Published:** 2021-03-17

**Authors:** Kai Fan, Chengying Lu, Gaofeng Shu, Xiu-Ling Lv, Enqi Qiao, Nannan Zhang, Minjiang Chen, Jingjing Song, Fazong Wu, Zhongwei Zhao, Xiaoling Xu, Min Xu, Chunmiao Chen, Weibin Yang, Jihong Sun, Yongzhong Du, Jiansong Ji

**Affiliations:** 1 Department of Radiology, Key Laboratory of Imaging Diagnosis and Minimally Invasive Intervention Research, School of Medicine, Lishui Hospital of Zhejiang University, Lishui, 323000 Zhejiang China; 2 Department of Radiology, Sir Run Shaw Affiliated Hospital, School of Medicine, Zhejiang University, Hangzhou, 310058 China; 3 Institute of Pharmaceutics, College of Pharmaceutical Sciences, Zhejiang University, Hangzhou, Zhejiang 310058 People’s Republic of China

**Keywords:** Magnetic resonance imaging, Superparamagnetic iron oxide, Polydopamine, Ferritin gene, Hepatocellular carcinoma

## Abstract

**Background:**

Hepatocellular carcinoma (HCC) is a common malignant tumor with poor prognosis. Magnetic resonance imaging (MRI) is one of the most effective imaging methods for the early diagnosis of HCC. However, the current MR contrast agents are still facing challenges in the early diagnosis of HCC due to their relatively low sensitivity and biosafety. Thus, the development of effective MR agents is highly needed for the early diagnosis of HCC.

**Results:**

Herein, we fabricated an HCC-targeted nanocomplexes containing SPIO-loaded mesoporous polydopamine (MPDA@SPIO), sialic acid (SA)-modified polyethyleneimine (SA-PEI), and alpha-fetoprotein regulated ferritin gene (AFP-Fth) which was developed for the early diagnosis of HCC. It was found that the prepared nanocomplexes (MPDA@SPIO/SA-PEI/AFP-Fth) has an excellent biocompatibility towards the liver cells. In vivo and in vivo studies revealed that the transfection of AFP-Fth gene in hepatic cells significantly upregulated the expression level of ferritin, thereby resulting in an enhanced contrast on T2-weighted images via the formed endogenous MR contrast.

**Conclusions:**

The results suggested that MPDA@SPIO/SA-PEI/AFP-Fth had a superior ability to enhance the MR contrast of T2-weighted images of tumor region than the other preparations, which was due to its HCC-targeted ability and the combined T2 contrast effect of endogenous ferritin and exogenous SPIO. Our study proved that MPDA@SPIO/SA-PEI/AFP-Fth nanocomplexes could be used as an effective MR contrast agent to detect HCC in the early stage.
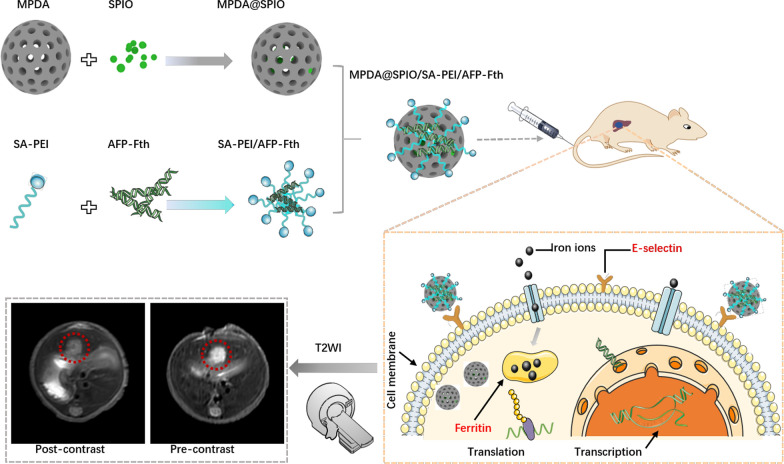

**Supplementary Information:**

The online version contains supplementary material available at 10.1186/s12951-021-00821-8.

## Background

Hepatocellular carcinoma (HCC) is a common malignant tumor, accounting for the third leading cause of cancer-related death worldwide [1]. The liver cancer patients suffer a poor prognosis, and the total five-year survive rate is below 20% [2, 3]. The high mortality and morbidity burden of HCC patients is largely resulting from the late presentation, and the early accurate detection of HCC is the most urgent challenges in clinical diagnosis. Magnetic resonance (MR) imaging is by far one of the most useful clinical diagnostic method for surveillance the patient at high risk [4]. Currently, all the gadolinium (Gd) based contrast agents are facing the shortcomings of low relativities, non-specific to target and quick excreted in vivo. On MR diagnostic examination, these drawbacks may hinder its application for accurate diagnosis of HCC.

Superparamagnetic iron oxide (SPIO) nanoparticle is a common used T2 contrast agent in the diagnosis of cancer, owing to its unique characteristics, such as a rapid response to an external magnetic field and great biocompatibility in vivo [5–8]. Due to the poor relaxivity, the simple utilization of SPIO as a contrast agent may result in inaccurate imaging or false-positive signals, such as blood pools, calcifications, and metal deposits [9–13]. Besides, the accumulation of SPIO in bones and brains caused by repeated administration and the inevitable ion leakage are potentially harmful for human being [14, 15]. Thus, the development of a novel SPIO-based contrast agent is extremely useful for the early diagnosis of HCC.

Nanotechnology is an effective strategy for delivering the contrast agents into the targeted tissue, which could not only result in an enhanced accuracy of diagnostic imaging but also a lower side effect. Recently, mesoporous polydopamine (MPDA) emerged as a novel nanocarrier has gained a growing attention in the drug delivery filed, which is due to its excellent biocompatibility, easy surface modification, and high photothermal conversion efficiency [16, 17]. Moreover, MPDA with mesoporous structures and larger surface areas is more efficient at encapsulating drugs to the conventional polydopamine (PDA) [18]. A recent study has also pointed that the T2-weighted MR contrast of bare SPIO could largely enhanced by incorporating them in the surface of MPDA, which therefore led to a more precise diagnosis of colon cancer [19]. In addition, it have been also found that PDA-based nanoparticles are effective at gene delivery [20–22].

In comparison with exogenous contrast agents, endogenous contrast agent has excellent native biocompatibility and biodegradability. In recent years, endogenous contrast agents such as, transferrin receptor, ferritin and β-galactosidase have attracted a considerable attention in the diagnosis of diseases [23–25]. Ferritin is an ubiquitous and conserved protein complex that consists of 24 protein subunits of ferritin heavy chain (Fth) and ferritin light chains, and it is regarded as the primary intracellular iron-storage protein. Fth is also the major regulator of ferritin activity, which displays ferroxidase activity to promote iron oxidation and chelation, thus could be used as an T2 weighted image (T2WI) endogenous contrast agent [26–32]. Previous studies have evidenced that the transfection of Fth reporter gene with alpha-fetoprotein promoter (AFP-Fth) could induce a high expression level of ferritin only in the liver cancer cells, which therefore shows a potential ability in the diagnosis of HCC via MRI technique in vivo [33]. However, the utilization of upregulated ferritin as an endogenous contrast agent alone is still not satisfactory for precise HCC diagnosis. Thus, the utilization of combined exogenous SPIO and endogenous ferritin as a dual-target T2-weighted MRI contrast agent might be a promising strategy for the effective diagnosis of HCC. Therefore, we deployed MPDA as a promising nanoplatform to co-delivery SPIO and Fth reporter gene for synergistic diagnosis of HCC.

The fabrication of nano-scaled contrast agent active targeting ability is also another way to reach the precise diagnosis of malignant tumors. It is well documented that E-selectin is an ideal target for drug delivery because it is highly overexpressed on the surfaces of inflammatory vascular endothelial cells (VECs) and tumor cells [34]. Sialic acid (SA) is highly distributed onto the surface of malignant cells and regard as a component of sialyl Lewisx antigen which participate in E-selectin binding [35, 36]. Our previous studies have found that sialic acid (SA) functionalized nanomedicines can specifically interact with E-selectin, thereby leading to a high accumulation of drugs in the tumor sites [37, 38]. Thus, the utilization of nano-agent with SA modification might be an effective approach for improving the accuracy of HCC diagnosis.

Herein, we developed an MRI-visible MPDA-based nanocomplexes incorporated with SA grafted polyethyleneimine (SA-PEI), SPIO and AFP-Fth (MPDA@SPIO/SA-PEI/AFP-Fth). MPDA loaded with SPIO was exploited as an exogenous T2 contrast agent and a nanodevice to deliver AFP-Fth gene into AFP positive HCC. In the presence of SA-PEI, AFP-Fth was protected from nuclease degeneration and escaped from endosomes via proton sponge effect [39], thus implemented as an endogenous contrast agent in T2WI. In addition, with the mediation of SA, MPDA@SPIO/SA-PEI/AFP-Fth could target to HCC and overexpress ferritin in hepatic tumor. The physicochemical characterization, cell uptake, cytotoxicity, and in vitro gene transfection ability of MPDA@SPIO/SA-PEI/AFP-Fth were investigated. The murine orthotopic HCC models were established, and the in vivo distribution and T2 weighted effect after the treatment of nanocomplexes was further investigated.

## Results and discussion

### Preparation and characterization of MPDA@SPIO nanoparticles

**Fig. 1:**
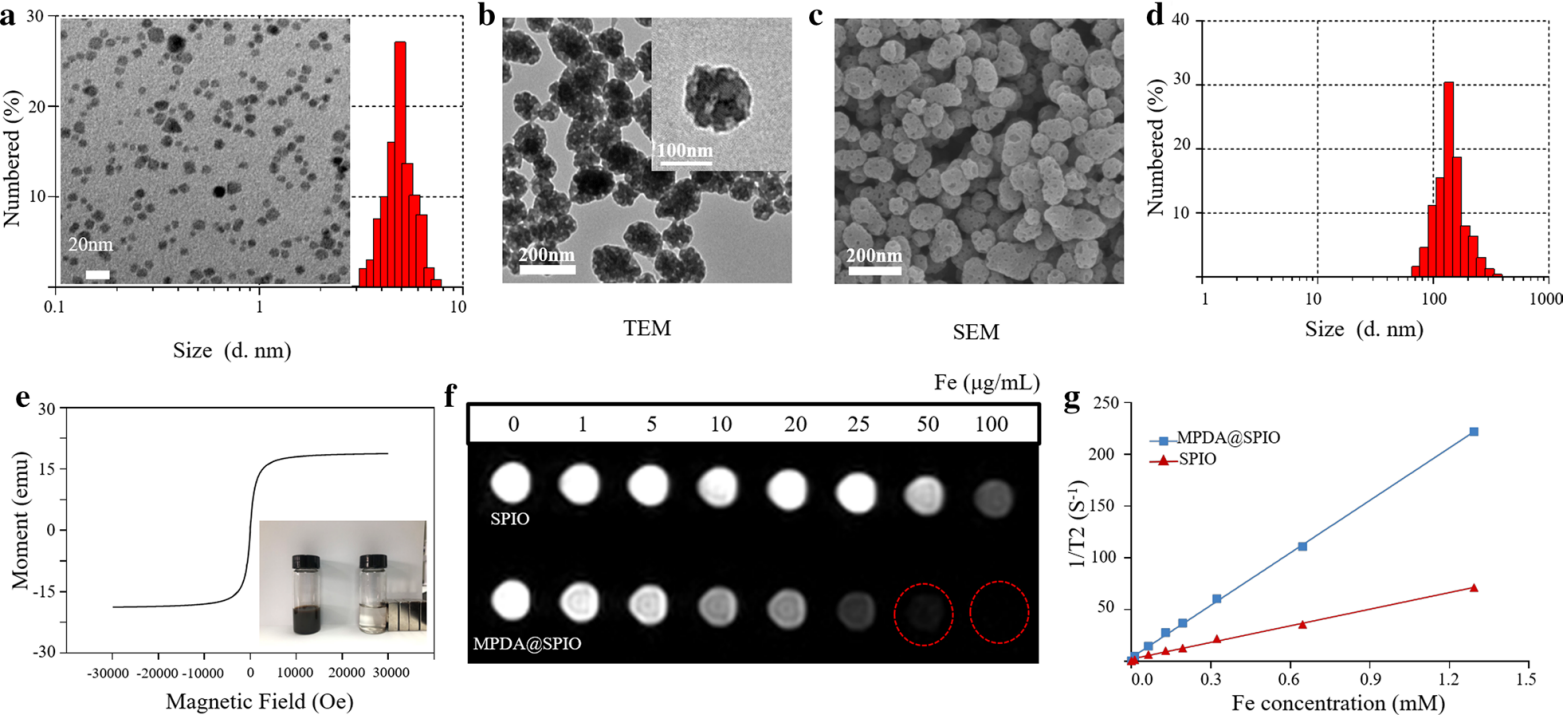
**a** TEM images and size distribution of SPIO. **b**–**d** TEM, SEM images and size distribution of MPDA@SPIO. **e** The M-H hysteresis curve of MPDA@SPIO and the Photograph of MPDA@SPIO without and with magnet. **f** T2 weighted images of free SPIO and MPDA@SPIO with different Fe concentrations **g**, and the corresponding T2 relaxation rates (1/T2, S^−1^)

In this part, SPIO nanoparticles were firstly synthesized by the classical thermal decomposition method with oleic acid (OA) as the surfactant. TEM and DLS measurements indicated that the prepared SPIO nanoparticles were narrowly distributed with particle diameters about 5.5 nm (Fig. 1a). MPDA@SPIO nanoparticles were then prepared according to the soft-template method [40]. As shown in Fig. 1b–d, MPDA@SPIO nanoparticles had well-defined mesoporous structures and spherical morphologies, and they were uniformly distributed with an average diameter of ~ 135 nm. A close examination of MPDA@SPIO nanoparticles showed that the SPIO were clearly incorporated inside the core of MPDA nanoparticles, suggesting the successful formulation of MPDA@SPIO nanoparticles in our study.

### Magnetic propriety of MPDA@SPIO nanoparticles

The magnetic ability is an important parameter to evaluate whether contrast agent is effective for MR imaging. The magnetic curves of MPDA@SPIO and free SPIO nanoparticles were detected by cycling the magnetic field from − 30 to + 30 kOe. As demonstrated in Fig. 1e, MPDA@SPIO exhibited a standard hysteresis loop and strong magnetism, which meant that MPDA@SPIO was superparamagnetic. In addition, MPDA@SPIO could also be attached by a magnet, which was due to the high saturation magnetization. To test the MR imaging properties, the T2-weighted images of MPDA@SPIO and free SPIO with different Fe concentrations were captured by 3.0- T MRI scanner (Fig. 1f). It revealed that the T2-weighted images of MPDA@SPIO and free SPIO changed from bright to dark black with increasing the Fe concentration. The T2 signal intensity of MPDA@SPIO decreased more obvious than that of free SPIO. The results in Fig. 1g showed that the proton transverse rate (1/T2) of MPDA@SPIO and free SPIO in T2WI had a linear relationship with the increment of Fe concentration. The T2 weighted MR intensity is prominent enhanced in MPDA@SPIO due to the high aggregation of SPIO core in MPDA, giving R2 relaxivity of 169 mM^−1^ s^−1^, which is ~ 3.2 times higher than the free SPIO (53.6 mM^−1^ s^−1^) and higher than that of commercial MRI contrast agent (Resvoist^®^ 130 mM^−1^ s^−1^; Sinerem^®^ 79 mM^−1^ s^−1^). These results suggested the MPDA@SPIO nanoparticles could be used as a potential T2 contrast agent with fewer injection dose in MRI diagnosis.

### Preparation and characterization of MPDA@SPIO/SA-PEI/AFP-Fth

**Fig. 2:**
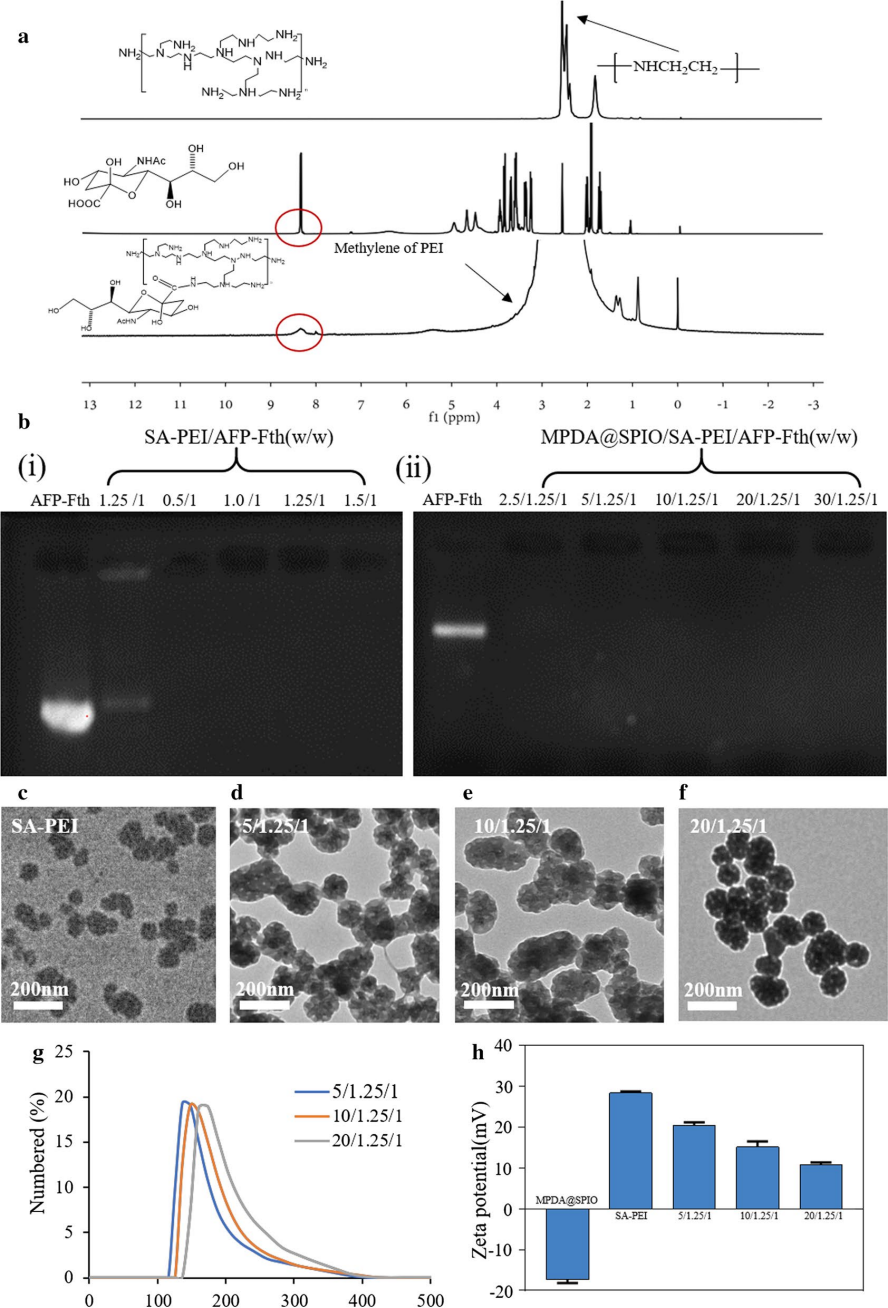
**a** ^1^H-NMR of SA, PEI, and SA-PEI, **b** Gel electrophoresis of SA-PEI/AFP-Fth (0.25, 0.5, 1.0, 1.25, 1.5:1, w/w), (MPDA@SPIO/SA-PEI/AFP-Fth = 2.5, 5, 10, 20, 30:1.25:1, w/w/w) nanocomplexes and free AFP-Fth. (**c**; **d**–**f**) TEM images and particle size distribution of MPDA@SPIO/SA-PEI/AFP-Fth (5; 10; 20/1.25/1 w/w). **d** Zeta potential of MPDA@SPIO, SA-PEI/AF-Fth (1.25/1) and MPDA@SPIO/SA-PEI/AFP-Fth (5; 10; 20/1.25/1 w/w)

The sialic acid-grafted PEI (SA-PEI) was synthesized by the NHS-mediated amidation between the carboxylate groups on SA and the amine groups on PEI. The ^1^H-NMR spectrum of the prepared SA-PEI was displayed in Fig. 2a. It was revealed that SA-PEI had the characteristic peaks of SA (–NH–, ∼8.1 ppm) [41] and PEI (–CH_2_CH_2_NH–, 2.4–3.0 ppm), demonstrating that SA-PEI was successfully synthesized in our study. Then, SA-PEI/AFP-Fth polyplexes with different ratio of SA-PEI and AFP-Fth were prepared by simply mixing these two opposite charged polymers via vigorous vortex. Agarose gel retardation assay was performed to assess the gene complexation ability of nanocomplexes (SA-PEI/AFP-Fth and MPDA@SPIO/SA-PEI/AFP-Fth). As displayed in Fig. 2b–i, the AFP-Fth could be well retarded by SA-PEI when the weight ratio (SA-PEI:AFP-Fth) was above 0.5. Previous studies have proved that PEI/DNA polyplexes with a mass ration of ~ 1.25 (nitrogen/phosphate molar ratio about 10) has optimal gene transfection [42]. Thus, the SA-PEI/AFP-Fth polyplexes with a fixed mass ratio of 1.25 was used for the following study. DLS results suggested that SA-PEI/AFP-Fth polyplexes prepared at this condition were uniformly distributed, and they had spherical particle with average size of around 105 nm (Fig. 2c).

MPDA@SPIO/SA-PEI/AFP-Fth nanocomplexes was formulated by mixing the MPDA@SPIO and SA-PEI/AFP-Fth together via electrostatic interaction. The physicochemical characteristics of MPDA@SPIO/SA-PEI/AFP-Fth prepared with different MPDA@SPIO:SA-PEI:AFP-Fth ratio were summarized in Table 1. There prepared MPDA@SPIO/SA-PEI/AFP-Fth showed certain increase in the particle size from 144.98 to 168.17 nm as the MPDA@SPIO:SA-PEI:AFP-Fth ratio was increased from 5:1.25:1 to 20:1.25:1. However, the surface charges of the corresponding MPDA@SPIO:SA-PEI:AFP-Fth showed the opposite tendency from 20.40 to 10.87 mV. In addition, all the samples showed a narrow polydispersity of < 0.3. We also observed that the MPDA@SPIO/SA-PEI/AFP-Fth prepared with a ratio of > 20:1.25:1 were highly unstable with large aggregation in a short incubation time, which was due to the reduced repulsive force between nanoparticles (data not shown). TEM examination indicated that the morphology of MPDA@SPIO/SA-PEI/AFP-Fth prepared with different MPDA@SPIO:SA-PEI:AFP-Fth ratio were similar (Fig. 2d–f). Gel retardation assay was also carried out to investigate the ability of MPDA@SPIO/SA-PEI/AFP-Fth to condense AFP-Fth into nanoparticle (Fig. 2b–ii). The results suggested the band of migrated AFP-Fth disappeared completely in MPDA@SPIO/SA-PEI/AFP-Fth complexes, indicating a complete DNA complexation regardless of the ratio of MPDA@SPIO:SA-PEI:AFP-Fth used in our study.

**Table 1:** Physicochemical characterization of the formulated nanoparticles

Sample	Mass ratio	Size (nm)	Polydispersity Index (PI)	Zeta potential (mv)
MPDA@SPIO		135.04 ± 6.4	0.229 ± 0.292	− 17.27 ± 0.9
SA-PEI/AFP-Fth	0/1.25/1	105.04 ± 0.8	0.188 ± 0.058	28.4 ± 0.4
MPDA@SPIO/SA-PEI/AFP-Fth	5/1.25/1	144.98 ± 5.6	0.090 ± 0.043	20.40 ± 0.8
MPDA@SPIO/SA-PEI/AFP-Fth	10/1.25/1	148.66 ± 10.4	0.189 ± 0.027	15.10 ± 1.4
MPDA@SPIO/SA-PEI/AFP-Fth	20/1.25/1	168.17 ± 8.3	0.245 ± 0.007	10.87 ± 0.5

N_2_ adsorption/desorption isotherms of MPDA and MPDA@SPIO/SA-PEI/AFP-Fth were also observed. As shown in Additional file 1: Fig. S1, the BET measurement of MPDA exhibited a typical type-IV of isotherm curve, which confirmed the mesoporous structure. However, when being loaded with SPIO and AFP-Fth, a mixed isotherm was observed in MPDA@SPIO/SA-PEI/AFP-Fth, indicating the channel of mesoporous structure was changed in the presence of SPIO, SA-PEI and AFP-Fth, and the BET specific surface areas decreased from 177.1634 m^2^/g to 16.3538 m^2^/g. These results also implied that SPIO, SA-PEI and AFP-Fth occupied the mesoporous channels of MPDA and successful synthesis of MPDA@SPIO/SA-PEI/AFP-Fth.

The stability of MPDA@SPIO/SA-PEI/AFP-Fth nanocomplexes were evaluated by constantly observing its size distribution in water, PBS, DMEM, and PBS + 10% FBS solutions. Dynamic light scattering (DLS) analysis were employed to evaluate the size changes. It was found that the size of MPDA@SPIO/SA-PEI/AFP-Fth nanocomplexes was not significantly changed during 7 days of storage in these mediums (Additional file 1: Fig S2A), and the TEM image of MPDA@SPIO/SA-PEI/AFP-Fth nanocomplexes after 7 days of storage in PBS + 10%FBS solution, which demonstrated the monodispersed particles without sign of gathering (Additional file 1: Fig S2B). These results suggested the excellent physiological stability of MPDA@SPIO/SA-PEI/AFP-Fth nanocomplexes.

### Cytotoxicity of MPDA@SPIO and the nanocomplexes

**Fig. 3:**
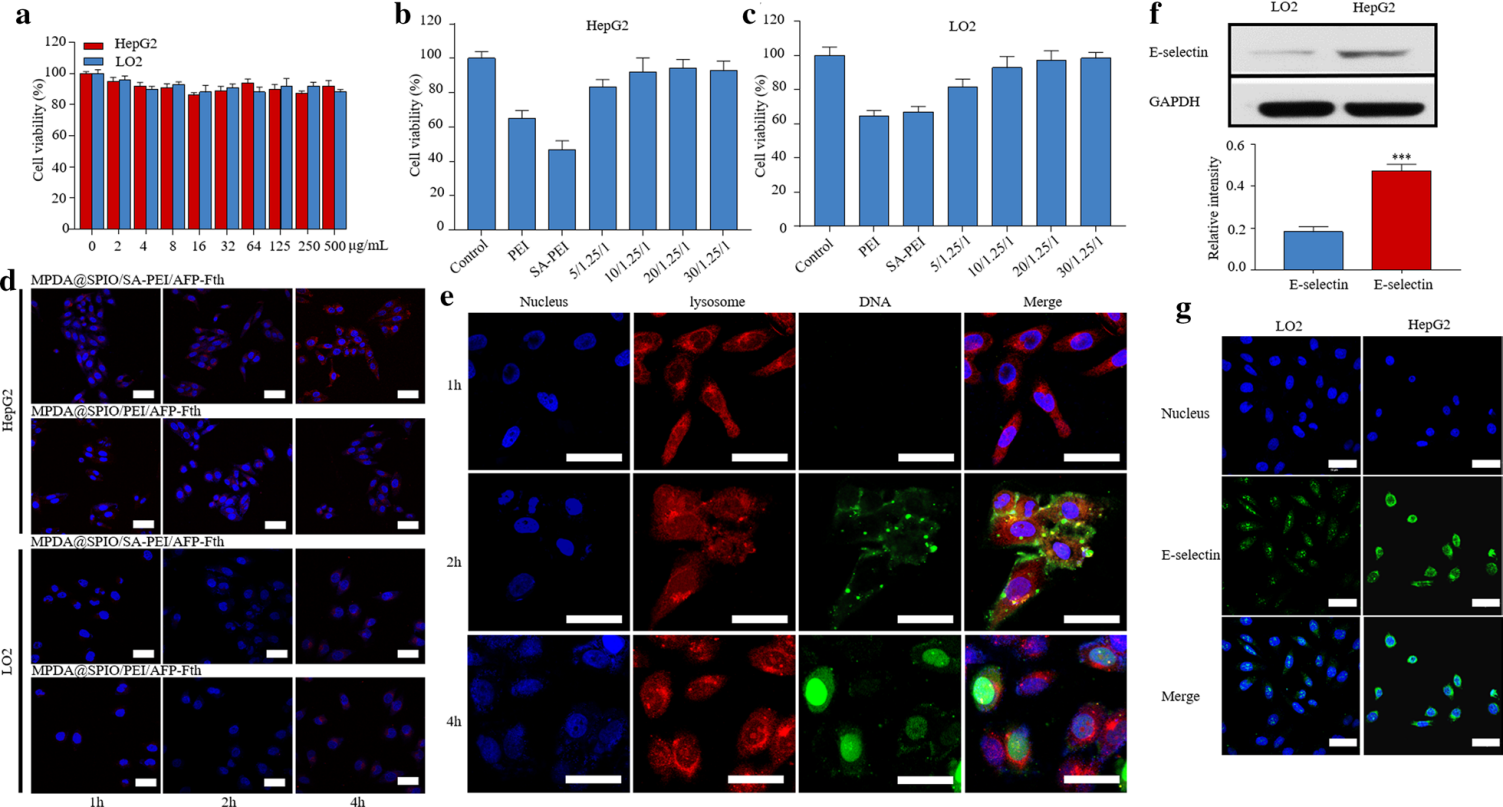
**a** Evaluation the cytotoxicity of different concentrations of MPDA@SPIO in HepG2 and LO2, (**b**, **c**) HepG2 and LO2 transfected by MPDA@SPIO/SA-PEI/AFP-Fth (0; 2.5; 5; 10; 20/1.25/1 w/w/w) and PEI/AFP-Fth (1.25/1 w/w/). **d** Confocal Laser Scanning Microscope (CLSM) images of HepG2 and LO2 cells incubated with the MPDA@SPIO/SA-PEI/AFP-Fth (20/1.25/1 w/w/w) labeled ICG for 1, 2, 4 h. **e** The FAM labeled DNA of MPDA@SPIO/SA-PEI/FAM-DNA internalized by HepG2 at 1, 2 and 4 h. **f** Western blot assay for E-selectin in HepG2 and LO2. **g** Fluorescence images of HepG2 and LO2 cells stained with E-selectin antibody. Scale bar = 50 μm. (n = 3; ***p < 0.001)

In vitro cytotoxicity of MPDA@SPIO and MPDA@SPIO/SA-PEI/AFP-Fth nanocomplexes transfected towards HepG2 and LO2 cells was performed by MTT assay. As shown in Fig. 3a, both of HepG2 and LO2 cells had cell viabilities of > 90% after they were treated MPDA@SPIO at a concentrations up to 500 μg/ml, suggesting the excellent biocompatibility of MPDA@SPIO nanoparticles[43, 44]. In Fig. 3b, c, it was observed that PEI/AFP-Fth and SA-PEI/AFP-Fth polyplexes exhibited a certain toxicity on LO2 and HepG2 cells, as evidenced by the cell viability was relatively low after transfection, and the targeting polyplexes showed a lower cell viability (SA-PEI/AFP-Fth; 46.9%) than the non-targeting one (PEI/AFP-Fth; 65.2%) in HepG2, which might be due to the higher transfection efficiency of the targeting one. In comparison, the viability of cells treated with MPDA@SPIO/SA-PEI/AFP-Fth in various mass ratios were much higher than that of PEI/AFP-Fth and SA-PEI/AFP-Fth, suggesting that the incorporation of MPDA@SPIO could largely reduce the cytotoxicity of SA-PEI/AFP-Fth. The possible reason behind this observation was that the MPDA@SPIO nanoparticles can immobilize the macromolecular chains, which leads to the decrease in the cytotoxicity of cationic polyplexes since lowered density of cationic residues [45, 46]. For example, PEI immobilized on PDA exhibited decreased cytotoxicity towards A549 cells [22]. Hence, the decreased cytotoxicity of MPDA@SPIO/SA-PEI/AFP-Fth nanocomplexes in the present work can also be ascribed to the decreased polyplexes cationic density on the MPDA@SPIO nanoparticles surfaces, exhibiting relatively low cytotoxicity and high biocompatibility for both tumor cells and normal cells.

### Targeting ability and cell uptake of MPDA@SPIO/SA-PEI/AFP-Fth in vitro

In this part, MPDA@SPIO/SA-PEI/AFP-Fth and MPDA@SPIO/PEI/AFP-Fth labeled with a fluorescent dye (ICG) were used for the cellular uptake study. The fluorescence images and intensity of HepG2 and LO2 cells treated with ICG-loaded MPDA@SPIO/SA-PEI/AFP-Fth and MPDA@SPIO/PEI/AFP-Fth for 1 h, 2 h, and 4 h, respectively. In Fig. 3d**,** it demonstrated that after treating with the nanocomplexes, both of HepG2 and LO2 cells showed an increase of fluorescence intensity with increasing the incubation time. However, it should be noted that the HepG2 cells treated with ICG-loaded MPDA@SPIO/SA-PEI/AFP-Fth exhibited the highest fluorescence intensity than the other three groups at the same incubation time, which indicated the targeting ability of MPDA@SPIO/SA-PEI/AFP-Fth towards the HepG2 cell. Besides, the iron content of treated groups was evaluated by ICP-MS. In the case of HepG2 cells, MPDA@SPIO/SA-PEI/AFP-Fth group showed a significant higher Fe content that of MPDA@SPIO/ PEI/AFP-Fth group at the same incubation time (Additional file 1: Fig S3). In comparison, the Fe content in the LO2 cells was not significantly different between MPDA@SPIO/SA-PEI/AFP-Fth and MPDA@SPIO/ PEI/AFP-Fth groups (Additional file 1: Fig S3). In addition, the results also suggested the internalization of MPDA@SPIO/SA-PEI/AFP-Fth into HepG2 cells was significantly higher than that of LO2 cells, because of the Fe content in HepG2 cells was much higher than that of LO2 cells. These results further confirmed MPDA@SPIO/SA-PEI/AFP-Fth has a specific targeting ability towards HepG2 cells.

In Fig. 3e, we also found that the fluorescence signal of FAM labeled DNA (FAM-DNA) was not appeared after 1 h of incubation. It may be due to the MPDA@SPIO nanoparticles was still absorbed and quenched the fluorescence of FAM-DNA [47]. After 2 h of incubation, the fluorescence signal of FAM-DNA (green color) was partially overlapped with that of lysosome (red color), and with the prolong of time at 4 h, the fluorescence signal of FAM-DNA was mainly distributed in the cell nucleus (blue color), suggesting that DNA could successfully escape from the lysosome and enter into the nucleus by MPDA@SPIO/SA-PEI/DNA nanocomplexes.

Previous studies have indicated that E-selectin is overexpressed on the surfaces of cancer cells, including the HepG2 cells [48]. And the upregulation of E-selectin in HepG2 cells was also confirmed in current work, where both of immunofluorescence and western blotting examination suggested that the expression level of E-selectin in HepG2 cells was much higher than that of LO2 cells (Fig. 3f, g). On the other hands, SA as a targeted molecule that could specifically interact with the E-selectin [8, 41]. Thus, the higher internalization ability of MPDA@SPIO/SA-PEI/AFP-Fth by HepG2 cells was closely related with its E-selectin targeting ability generated by the SA modification. These results clearly indicated that the specific binding and uptake of the MPDA@SPIO/SA-PEI/AFP-Fth nanocomplexes by HepG2 should be mediated by the E-selectin overexpressed on the cell surface, and MPDA@SPIO/SA-PEI/AFP-Fth could also deliver AFP-Fth to the nuclear of HepG2 after 4 h of incubation, which created the possibility of ferritin expression.

### The nanocomplexes transfection experiment in vitro

**Fig. 4:**
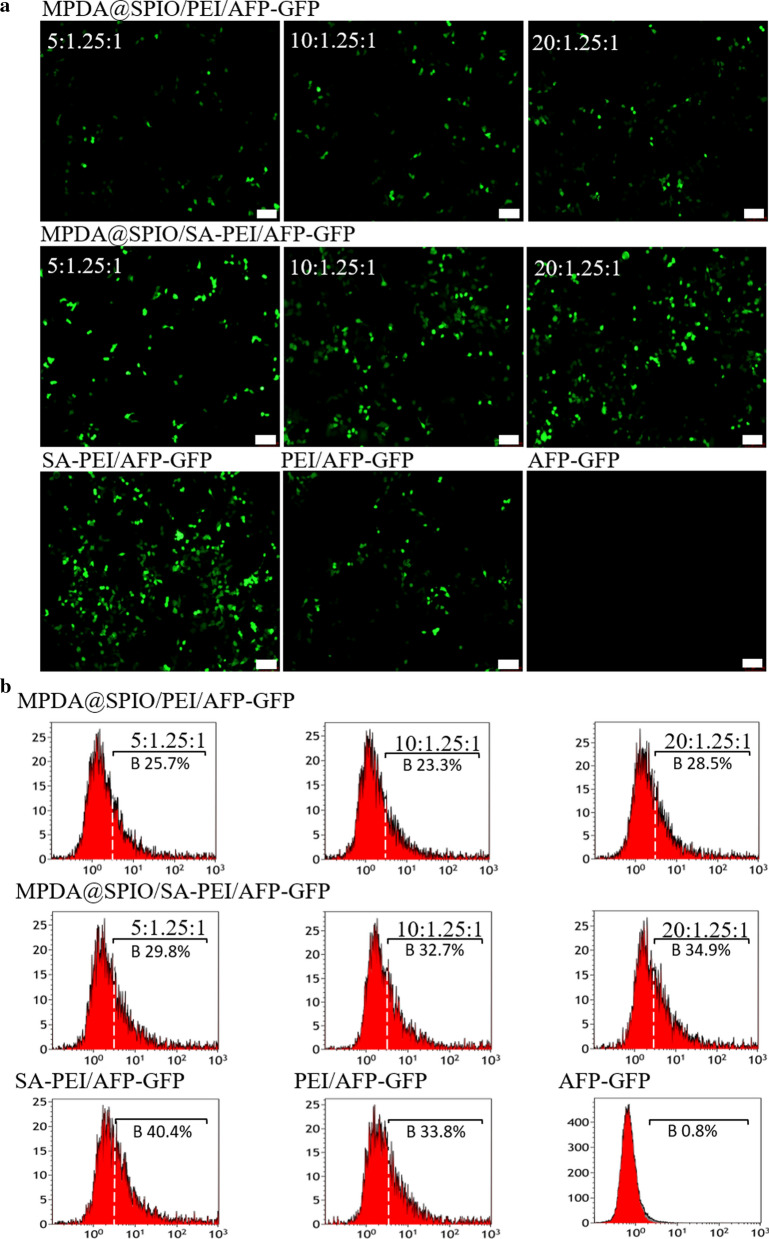
**a** GFP expression levels in HepG2 after transfected in different mass ratio of MPDA@SPIO/SA-PEI/AFP-GFP(5; 10; 20/1.25/1w/w/w), MPDA@SPIO/PEI/AFP-Fth (5; 10; 20/1.25/1 w/w/w), SA-PEI/AFP-GFP (1.25/1 w/w), PEI/AFP-GFP (1.25/1 w/w) and AFP-GFP was set as the negative group (bar = 100 μm). **b** Flow cytometry counts GFP expression rate in HepG2 after 48 h transfection

As nanocomplexes of MPDA@SPIO/SA-PEI/AFP-Fth were endowed with promising attributes like gene carrier ability and high uptake ability to HepG2, we next focus our attention on its role as gene delivery vehicle. Plasmid DNA encoding a green fluorescent protein gene with an AFP promoter (AFP-GFP) instead of AFP-Fth were used to prepare MPDA@SPIO/SA-PEI/AFP-GFP and MPDA@SPIO/PEI/AFP-GFP for the evaluation of in vitro gene transfection ability. The fluorescent images suggested that the treatment of MPDA@SPIO/SA-PEI/AFP-GFP prepared with different mass ratio resulted in a different expression level of GFP in HepG2 cells (Fig. 4a). This effect was also confirmed by flow cytometry assay, which showed that the GFP transfection efficiency of MPDA@SPIO/SA-PEI/AFP-GFP ranged from 29.8% to 34.9% with the MPDA@SPIO mass ratio from 5/1.25/1 to 20/1.25/1 (Fig. 4b). In addition, the fluorescent images and cytometry assay displayed that the transfection efficiency of MPDA@SPIO/SA-PEI/AFP-GFP was much higher than that of MPDA@SPIO/PEI/AFP-GFP at the same mass ratio. This result could be ascribed to the targeting ability of MPDA@SPIO/SA-PEI/AFP-GFP towards HepG2 cells. And the transfection efficiency of MPDA@SPIO/SA-PEI/AFP-GFP was also compared with that of PEI/AFP-GFP and SA-PEI/AFP-GFP. Flow cytometry assay revealed that the transfection efficiency of MPDA@SPIO/SA-PEI/AFP-GFP (20/1.5/1), PEI/AFP-GFP and SA-PEI/AFP-GFP was 34.9%, 33.8% and 40.4%, respectively. This result suggested that the incorporation of MPDA@SPIO did not significantly affect the transfection efficiency of SA-PEI/AFP-GFP, and MPDA@SPIO/SA-PEI/AFP-GFP (20/1.5/1) transfection efficiency was nearly the same with PEI/AFP-GFP (34.9%). Overall, the above findings indicated the optimal MPDA@SPIO/SA-PEI/AFP-GFP for gene transfection was those prepared with mass ratio of 20/1.5/1, which was therefore fixed at this mass ratio in the following studies.

### AFP-Fth expression ability of MPDA@SPIO/SA-PEI/AFP-Fth in HepG2 cells

**Fig. 5:**
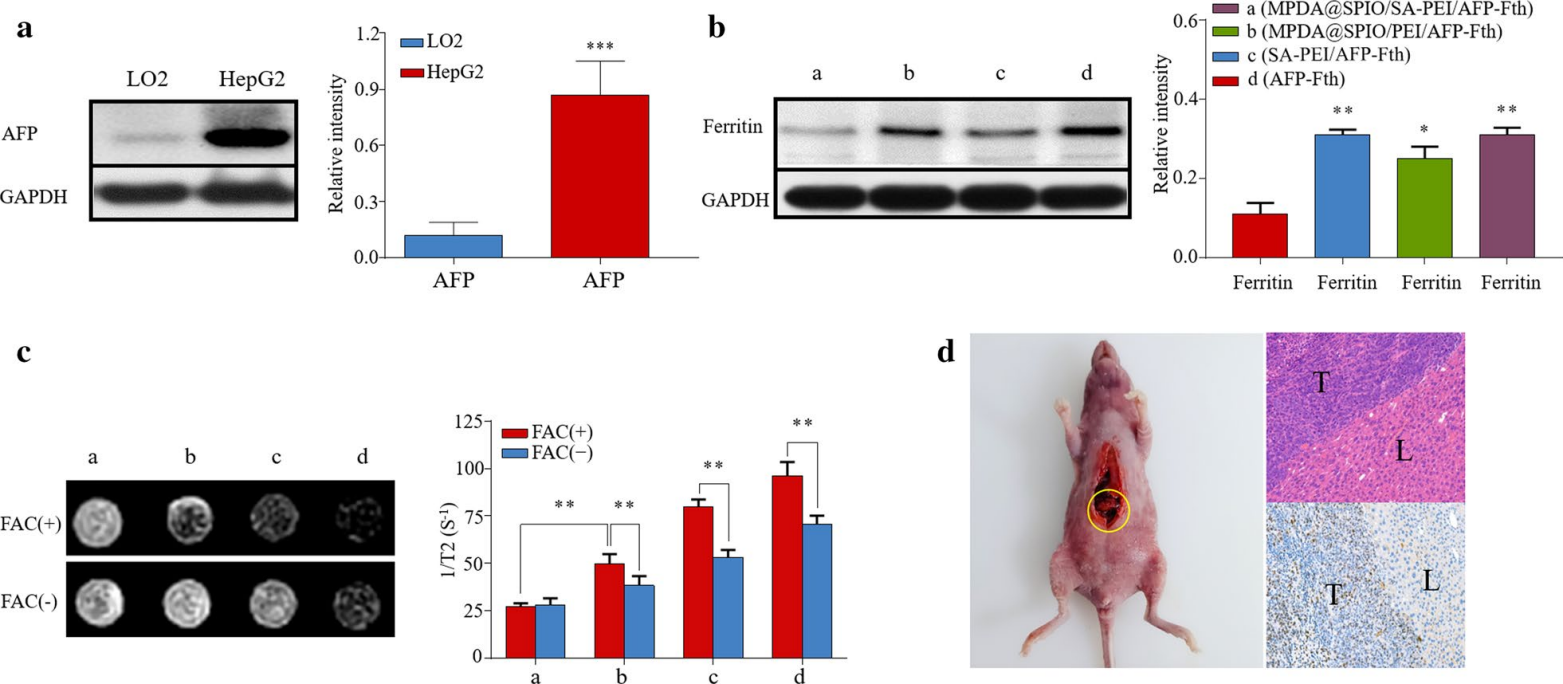
**a** Western blot study of AFP between HepG2 and LO2. **b** Ferritin level of HepG2 that transfected by MPDA@SPIO/SA-PEI/AFP-Fth, MPDA@SPIO/PEI/AFP-Fth, SA-PEI/AFP-Fth. Free AFP-Fth was also added as control group. **c** T2-weighted images of transfected HepG2 cells with or without FAC supplement, and the chart of 1/T2 (S^−1^) values. **d** The tumor model made in orthotopic injection HepG2 cells, the yellow cycle stands for tumor in liver. Microscope image (× 10) of HE and Ki-67 immunofluorescence stained liver tissue section, “L” indicated the normal liver, “T” indicated the tumor tissue. (**a**, MPDA@SPIO/SA-PEI/AFP-Fth **b**, MPDA@SPIO/PEI/AFP-Fth **c**, SA-PEI/AFP-Fth d, AFP-Fth) (n = 5; ***p < 0.001, **p < 0.01, *p < 0.05, compared with untreated cells)

First of all, the expression level of AFP in HepG2 and LO2 cells were investigated by western blotting assay. As shown in Fig. 5a, the HepG2 cells expressed much higher level of AFP relative to the LO2 cells. This result also indicated that AFP-Fth could only be transfected in the AFP positive HepG2 cells. Then, the change in the expression levels of ferritin in HepG2 cells was evaluated after 48 h post transfection with MPDA@SPIO/SA-PEI/AFP-Fth, MPDA@SPIO/PEI/AFP-Fth, SA-PEI/AFP-Fth, and HepG2 treated with free AFP-Fth was set as control group. It was found that the treatment of free AFP-Fth had little impact on the expression level of ferritin (Fig. 5b). In comparison, there was a considerably enhanced expression level of ferritin in HepG2 cells after they were treated with MPDA@SPIO/SA-PEI/AFP-Fth, MPDA@SPIO/PEI/AFP-Fth or SA-PEI/AFP-Fth. More precisely, MPDA@SPIO/SA-PEI/AFP-Fth and SA-PEI/AFP-Fth induced a similar expression level of ferritin, which was much higher than that of MPDA@SPIO/PEI/AFP-Fth. These results were consistent with the results of GFP expression levels, which was due to that the targeted molecule of SA provided the nanoparticles with an enhanced cellular accumulation. According to the previous reports, Fe supplement is indispensable to enable iron chelation by ferritin [32, 48]. Herein, ferric ammonium citrate (FAC) was chosen to add in the current work to elevate the free iron during gene transfection experiment. To confirm the role of AFP-Fth and SPIO could be worked as an endogenous and exogenous contrast agent for MR T2 weighted detecting, MPDA@SPIO/SA-PEI/AFP-Fth, MPDA@SPIO/PEI/AFP-Fth and SA-PEI/AFP were used to transfect HepG2 cells respectively, and the untreated HepG2 was set as control group. T2-weighted images of HepG2 cells were obtained and the signal intensity of cells were quantified in Fig. 5c. It demonstrated that, in the existence of FAC, MPDA@SPIO/SA-PEI/AFP-Fth, MPDA@SPIO/PEI/AFP-Fth and SA-PEI/AFP-Fth could significantly decrease the MR signal intensity of HepG2 cells than non-supplement group (p < 0.01). Moreover, the MR signal intensity of HepG2 cells transfected by MPDA@SPIO/SA-PEI/AFP-Fth showed more obviously decreasing trend than that of MPDA@SPIO/PEI/AFP-Fth, which might be due to the targeting ability of SA. HepG2 cells transfected by SA-PEI/AFP-Fth with FAC exhibited a significant decrease of T2 signal intensity than that of absence of FAC (p < 0.01). The outcome was consisted with our previous study that the intracellular overexpressed ferritin in transfected cells could chelate with free iron in the presence of FAC, and further decrease the T2 relaxation time [33]. No significant difference was observed in control group after adding with FAC, as demonstrated by the similar signal intensity values. These results suggested that HepG2 cells treated with MPDA@SPIO/SA-PEI/AFP-Fth can greatly enhanced T2WI by synergistic AFP-Fth expression and MPDA@SPIO internalization.

### In vivo MR imaging effects

The orthotopic hepatoma murine models were built, and HepG2 tumor cells were observed to be orthotopically developed in the liver of mice (Fig. 5d). The tumor histopathology was evaluated by H&E staining, it showed a resembled poorly differentiated human HCC cells with larger, pleomorphic and bigger nucleus, and also had abundant cytoplasm, which confirmed the orthotopic hepatoma murine models were successfully established.

**Fig. 6:**
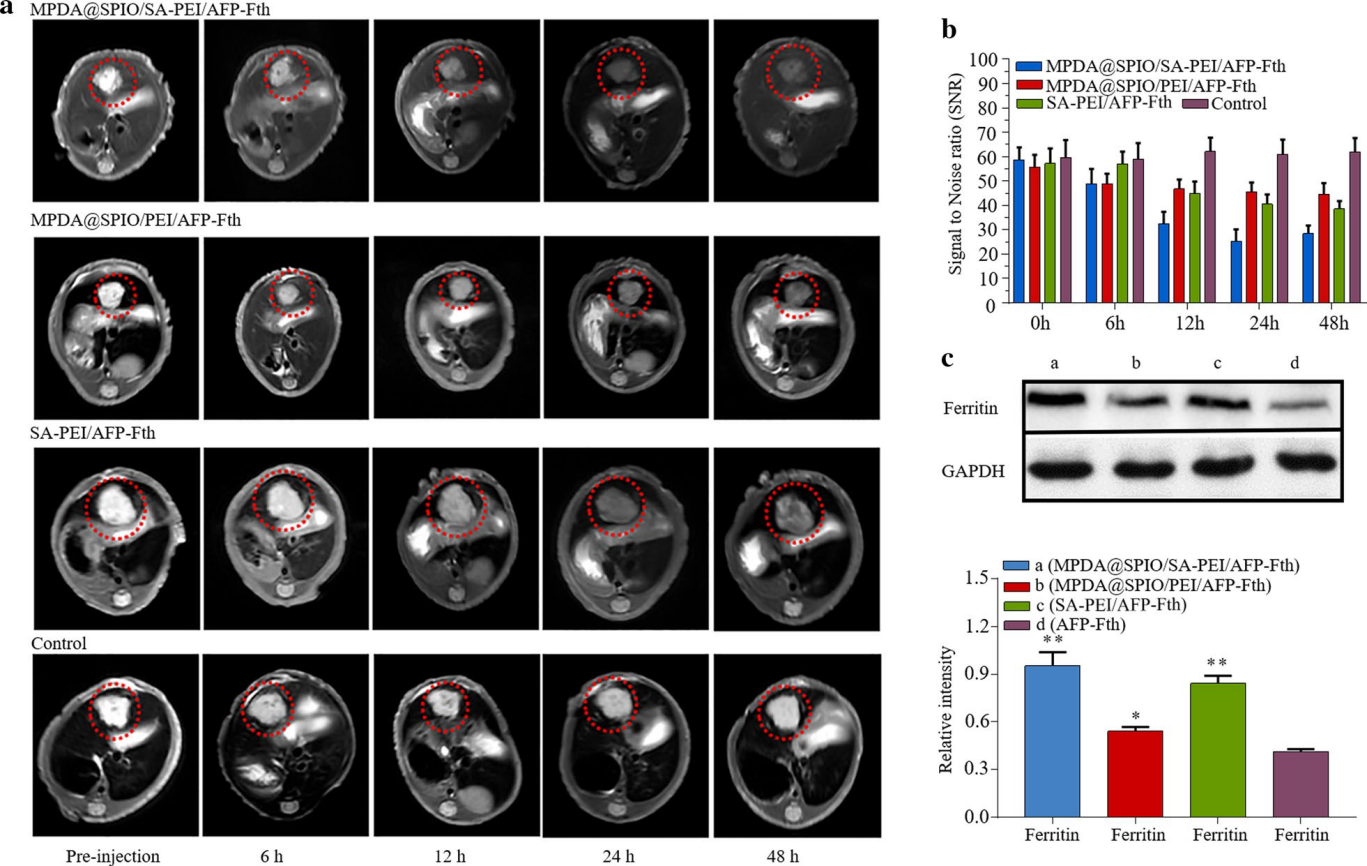
**a** In vivo evaluation of the T2 weighted images of nude mice bearing orthotopic HCC tumor at before and after administration of MPDA@SPIO/SA-PEI/AFP-Fth, MPDA@SPIO/PEI/AFP-Fth, SA-PEI/AFP-Fth for 6, 12, 24, 48 h respectively, and untreated mice were scanned as control group. The red dash cycle indicated the tumor site. **b** MRI SNR of tumor site in different groups at pre-injection and 6, 12, 24, 48 h of post-injection. **c** The expression of ferritin in MPDA@SPIO/SA-PEI/AFP-Fth, MPDA@SPIO/PEI/AFP-Fth, SA-PEI/AFP-Fth and control group. (n = 3; *p < 0.05; **p < 0.01)

BALB/c mice bearing orthotopic HepG2 tumors were used to evaluate the T2WI performance of MPDA@SPIO/SA-PEI/AFP-Fth for cancer diagnosis. The mice were tail-intravenously injected with the contrast agents of MPDA@SPIO/SA-PEI/AFP-Fth, MPDA@SPIO/PEI/AFP-Fth and SA-PEI/AFP-Fth respectively, and the mice treated with PBS were set as the control group. Both the T2-weighted images and T2 SNR of HepG2 liver tumors at before and 6, 12, 24, 48 h after tail vein injections were acquired (Fig. 6a, b).

Clearly, after injection of either MPDA@SPIO/SA-PEI/AFP-Fth or MPDA@SPIO/PEI/AFP-Fth, the T2-weighted images of the tumors became dark at 6 h, the tumor SNR showed no obvious difference between MPDA@SPIO/SA-PEI/AFP-Fth and MPDA@SPIO/PEI/AFP-Fth, which was mainly due to the fact that the nanocomplexes could be passive accumulated in the tumor through EPR effect [8]. However, from 6 to 12 h, the tumor SNR of the mice treated with MPDA@SPIO/SA-PEI/AFP-Fth were largely decreased from 48.8 to 32.3, while there was much less change in the group of MPDA@SPIO/PEI/AFP-Fth (48.9–46.6). At 12 to 48 h post-injection, the tumor SNR of the mice treated with MPDA@SPIO/PEI/AFP-Fth has been always stayed at about a same level (44.6–46.7). In contrast, the tumor SNR of the mice that treated with MPDA@SPIO/SA-PEI/AFP-Fth still weakened at 12–24 h post-injection and the tumor section achieved superior darkness at 24 h (SNR = 25.2). It may ascribe to the active target ability of SA, which made the nanocomplexes internalized by tumor cells that overexpressed E-selectin and retained in the tumor lesion. In another group of SA-PEI/AFP-Fth, the tumor SNR began to decrease at 12 h of post-injection and gradually enhanced in the rest time (from 44.9 to 38.5). It illustrated that the Fth reporter gene with AFP promoter can be worked for T2 weighed imaging when the ferritin expression reached at sufficient level required for a measurable of T2WI effect. Compared with the SA-PEI/AFP-Fth group, the combination of AFP-Fth and MPDA@SPIO in MPDA@SPIO/SA-PEI/AFP-Fth yielded more prominent contrast effect from 24 to 48 h, which sustained the tumor SNR in a relative low level (25.3 to 28.3). The enhancement of tumor imaging by synergistic effect through exogenous and endogenous way was consistent with the findings in the previous studies in vitro.

It can be concluded from these results that the nanocomplexes modified with an active targeting was more sensitive and effective than passive targeting, meanwhile long-term T2 weighted imaging effect was acquired by combining the AFP-Fth that selectively expressed in HCC, which was also helpful for observation of the tumor progression.

To further testify the MPDA@SPIO/SA-PEI/AFP-Fth brought about the overexpression of ferritin in tumor, the mice were sacrificed after the MRI study, and the tumor tissues were collected. Western blot tests were performed after the processing of the specimens (Fig. 6c). The group of MPDA@SPIO/SA-PEI/AFP-Fth and SA-PEI/AFP-Fth exhibited statistically significant improvements of the ferritin expression compared with the control group (p < 0.01). Thereinto, the expression level of ferritin in the MPDA@SPIO/SA-PEI/AFP-Fth, MPDA@SPIO/PEI/AFP-Fth and SA-PEI/AFP-Fth was significantly higher than that in the MPDA@SPIO/PEI/AFP-Fth treatment group (p < 0.05).

**Fig. 7:**
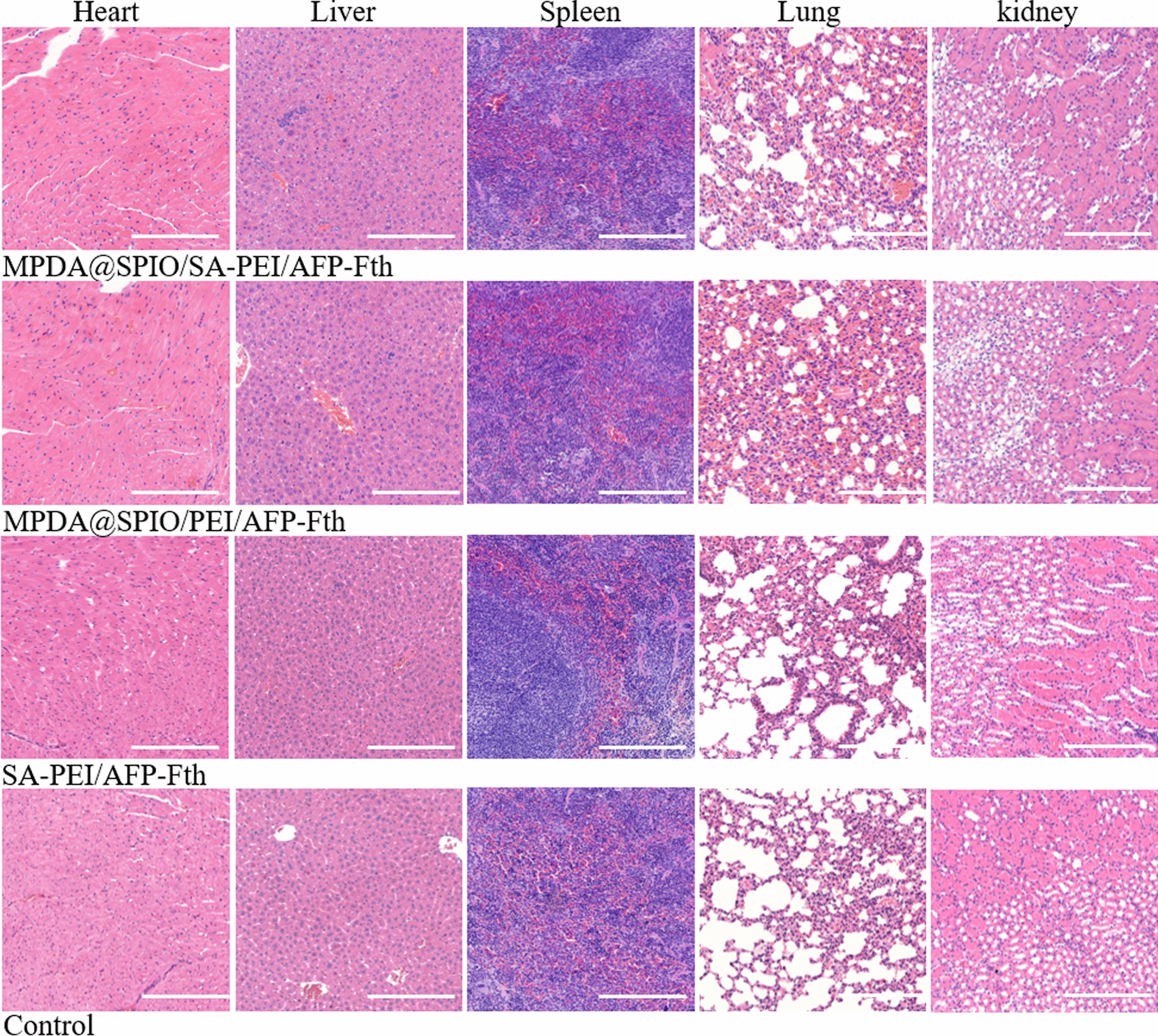
H&E-stained images of major organs exteriorized from the healthy control mice and MPDA@SPIO/SA-PEI/AFP-Fth, MPDA@SPIO/PEI/AFP-Fth, SA-PEI/AFP-Fth injected mice 48 h after treatments. Scare bar = 200 μm

Finally, we evaluated the histocompatibility of MPDA@SPIO/PEI/AFP-Fth on nude mice after 48 h of injection. Major organs of the treated mice were excised and stained by H&E staining (Fig. 7). Heart, liver, spleen, lung and kidneys of all the groups after treatment exhibited normal physiological morphologies without significant organ damage or significant abnormalities. These preliminary results demonstrated the biosafety on mice at the dose used in the present study.

## Conclusions

In this investigation, MPDA@SPIO/SA-PEI/AFP-Fth nanocomplexes as a novel diagnosis agent for targeting hepatoma cells was synthesized. The nanocomplexes possessed favorable magnetic properties and good biosafety. It demonstrated a dual targeting strategy that combined active targeting of SA with the plasmid that consisted AFP promoter, which was propitious accumulated and expressed the report gene in hepatoma cells. Both in vitro and in vivo studies revealed that MPDA@SPIO/SA-PEI/AFP-Fth had excellent targeting ability and transfection ability towards hepatic cancer. Owing to the combination of MPDA@SPIO and the overexpression of ferritin, the nanocomplexes could be functioned as a best T2 contrast agent compared to other groups in the orthotopic HCC mice. The effective of darkening tumor of HCC under T2WI model enable highly sensitive diagnosis of HCC in a long-term surveillance. On this basis, we hoped that this dual target nanocomplexes will further promote the development of next-generation clinical MRI contrast agents with exogenous and endogenous contrast enhancement for tumor diagnosis.

## Methods

### Materials

Iron acetylacetonate (Fe(acac)_3_, 99%), 1,2-dihydroxydodecane (90%), benzyl ether (99%), oleyamine (OLA, 70%), oleic acid (OA, 90%), dopamine hydrochloride (DA, 99.0%), Sialic acid (SA) and 3-(4,5-dimethylthiazol-2-yl)-2,5-diphenyltetrazolium bromide (MTT) were purchased from Aladdin Inc (Shanghai, China). Pluronic P123, F127, 1,3,5-trimethyl benzene (TMB), Indocyanine Green (ICG), carbodiimide (EDC), N-hydroxy succinimide (NHS) and Branched PEI (25 kDa) were purchased from Sigma-Aldrich Inc. (St. Louis, MO, USA). Ammonia solution (25–28%) and ethanol were purchased from Sinopharm Chemical Reagent Co., Ltd (Shanghai, China). Methoxy-poly (Ethylene Glycol) Amine (mPEG-NH2; Mw5000) was purchased from ToYongBio Tech.Inc. (Shanghai, China). DAPI (1:1000) and Lyso-Tracker Red purchased from Beyotime Co., Ltd, (Shanghai China). Primary antibodies including anti-AFP, anti-ferritin heavy chain and Anti-CD62E (E-selectin) antibodies were purchased from Abcam (Abcam, Cambridge, U.K., ab65080). The 6001 bp plasmid encoding ferritin heavy chain (h-Fth), the 4.7-kb plasmid encoding green fluorescence protein (pGFP-N1), GV349 vector were purchased from GeneChem Co., Ltd (Shanghai, China). Plasmid Miniprep Kit was purchased from Invitrogen (Carlsbad, USA).

HepG2 and LO2 cells were purchased from Cell Bank of Chinese Academy of Sciences, (Shanghai, China). The cells were cultured in Dulbecco’s modified Eagle’s medium (DMEM) with high glucose, 5% fetal bovine serum (Gibco, USA) and 1% penicillin–streptomycin in a 37 ℃ incubator with 5% CO_2_.

BALB/c nude mice (4 weeks; body weight:18 ± 2 g) were purchased from the Silaike Laboratory Animal Co., Ltd (Shanghai, China).

### Synthesis SPIO-loaded mesoporous polydopamine nanoparticles (MPDA@SPIO)

Oleic acid (OA) modified SPIO were synthesized following the previous classical thermal decomposition method [49]. Oleic-acid-stabilized SPIO was redispersed in TMB at a concentration of 5 mg/mL. To synthesize SPIO loaded-mesoporous polydopamine nanoparticles (MPDA@SPIO), 30 mg P123, 75 mg F127, and 150 mg dopamine hydrochloride were co-dissolved in 10 ml 40% ethanol solution. After stirred for 10 min, 0.4 ml TMB containing SPIO as-prepared before was inject into the above mixture and followed by ultrasonic treatment (Sonicator JY92-II DN, China) for 10 min to form a microemulsion. Then 0.375 ml of ammonium hydroxide was dropped in the microemulsion and vigorous stirred for 4 h at the room temperature. Finally, the products were collected by centrifugation (14000 rpm for 20 min) and washed with DI water and ethanol for serval times followed by lyophilization evaporation then stored at 4 ℃ for further use. To prepare MPDA, the TMB without SPIO was used instead of SPIO containing TMB, while the other procedures were the same as MPDA@SPIO preparation.

### Preparation of MPDA@SPIO/SA-PEI/AFP-Fth

Firstly, SA-PEI was synthesized by conjugated the amino groups of PEI and carboxyl groups of SA with acylamino links. Typically, 0.03 g SA, 0.07 g NHS, and 0.01 g EDC were co-dissolved in a flask, stirring at 50 ℃ for 1 h to activate SA, then 2.5 mg PEI were added. The mixture was stirred at 60 °C for another 24 h. After the reaction, the resultant product was collected and dialyzed for 48 h, then lyophilized and stored in a cool and dark place. The structure confirmation of SA-PEI was determined via ^1^H-NMR spectra (AC-80, Bruker Bios pin. Germany). Secondly, the polyplexes was spontaneous yielded through SA-PEI and the plasmid (AFP-Fth) with the mass ratio of 1.25/1 (SA-PEI:AFP-Fth = 1.25, w/w). Thirdly, different mass of MPDA@SPIO were mixed with polyplexes as-prepared before, followed by vortex for 5 s and 30 min incubation at room temperature. MPDA@SPIO/SA-PEI/AFP-Fth nanocomplexes (MPDA@SPIO:SA-PEI:AFP-Fth = 5, 10, 20, 30:1.25:1, w/w/w) in various mass ratio of MPDA@SPIO to AFP-Fth were prepared. For the control groups, MPDA@SPIO combined with PEI condensed AFP-Fth (MPDA@SPIO/PEI/AFP-Fth) and SA-PEI condensed AFP-Fth (SA-PEI/AFP-Fth) were prepared with the similar way.

### Construction of plasmids

To synthesize AFP-Fth, PCR amplification and standard cloning techniques were used to insert Fth gene from h-Fth into the linearized vector (GV349). For PCR amplifications, different 5′ and 3′ end primers were used to generate the fusion vectors. The double digested PCR product was ligated into BamHI/KpnI, digested GV349 with T4-DNA ligase and the ligation mixture was transformed into competent cells and plated on LB with Kanamycin for selection. The obtained antibiotic-resistant colonies were screened by colony PCR, and the plasmids were verified by sequencing after the miniprep. The same way to construct the plasmid that express enhanced green fluorescence protein with AFP promoter (AFP-GFP).

### Physicochemical characterization of MPDA@SPIO and MPDA@SPIO/SA-PEI/AFP-Fth

The hydrodynamic diameters, polydispersity index (PI) and zeta potential of the MPDA@SPIO nanoparticles, cationic polyplexes and MPDA@SPIO/SA-PEI/AFP-Fth were measured by a Zetasizer (3000HS, Malvern Instruments Ltd, UK). The morphologies of the nanoparticles were observed by the transmission electronic microscopy (TEM, JEM-1200EX, JEOL, Japan) and scanning electron microscopic (SEM, Gemini SEM 300, Germany). Magnetic measurements were performed by saturated magnetic intensity using a vibrating sample magnetometer (PPMS, Quantum Design, MPMS-XL-5, CA, USA) at 300 K by cycling the magnetic field between − 30 and + 30 kOe. Free SPIO and MPDA@SPIO aqueous diluted at different concentration gradients in various SPIO concentrations (0, 1, 5, 10, 20, 25, 50, 100 μg/ml) and scanned by 3.0-T MRI Scanner (Philips, INGENIA 3.0 T) in T2-weighted fast spin echo sequence (TR = 3000 ms, TE = 80 ms, matrix = 256 × 192, FOV = 120 mm × 90 mm, side thickness = 1.5 mm) to get T2-weighted images and signal intensity value. The transverse relaxivity (r2) was calculated through the curve-fitting of the inverse transverse relaxation time (1/T2; S^−1^) versus the SPIO concentration. N_2_ adsorption/desorption isotherm curves were recorded by a surface area and porosimetry system (Micromeritics, HD88, USA). The BET method was used to evaluate the specific surface areas of MPDA and MPDA@SPIO/SA-PEI/AFP-Fth.

### Gel electrophoresis experiment

The SA-PEI/AFP-Fth polyplexes (0.25, 0.5, 1.0, 1.25, 1.5:1, w/w), MPDA@SPIO/SA-PEI/AFP-Fth nanocomplexes (2.5, 5, 10, 15, 20, 30:1.25:1, w/w/w) and free AFP-Fth were incubated at 37 ℃ in DEPC solutions and the DNA movement at 100 v was detected by gel retardation experiment. SA-PEI/AFP-Fth and MPDA@SPIO/SA-PEI/AFP-Fth nanocomplexes and free AFP-Fth were electrophoresed on a 1% agarose gel containing nucleic acid dye for 30 min, then the gels were stained with Ethidium Bromide (EtBr) and visualized on a UV transilluminator (GL 200, Kodak, Windsor, CO, USA).

### Cytotoxicity of MPDA@SPIO and MPDA@SPIO/SA-PEI/AFP-Fth

Cytotoxicity of MPDA@SPIO nanoparticles and MPDA@SPIO/SA-PEI/AFP-Fth nanocomplexes were evaluated by MTT assay. Briefly, HepG2 and LO2 cells were seeded to 96-well plates (5 × 10^3^ cells/well) incubating for 24 h and subsequently added with various of concentrations of MPDA@SPIO and continue to culturing for 48 h. MPDA@SPIO/SA-PEI/AFP-Fth in various mass ratios (5, 10, 20:1.25:1, w/w/w) were dispersed in DMEM and cultivated with HepG2 and LO2 cells for 4 h respectively, the amount of AFP-Fth was fixed at 0.1 μg/well, then the medium removed and replaced with the serum-containing medium and kept for 48 h. After the incubation, the cell viabilities were measured by MTT assay. Briefly, 20 µl MTT solution (5 mg/mL) was added to each well for 4 h, and DMSO was added to dissolve formazan crystals via shaking slowly (90 rpm, 37 °C, 30 min). The absorption was measured at 570 nm using a microplate spectrofluorometer (Bio-Rad, model680, USA). Cytotoxicity of SA-PEI/AFP-Fth (1.25:1, w/w) and PEI/AFP-Fth (1.25:1) were evaluated by the similar way.

### Cellular uptake study

Indocyanine green (ICG) was chosen to be attached on the surface of MPDA@SPIO resulting in the formation of ICG-MPDA@SPIO and detected using confocal laser scanning microscopy (CLSM, OLYMPUS IX83-FV3000-OSR). The synthesis of ICG-MPDA@SPIO was according to the previous report [50]. In brief, MPDA@SPIO and ICG were co-dissolved in weak acid aqueous solution (pH 3.5–5.5) stirred for 4 h in the room temperature, after centrifugation the resultant product was redispersed in DI water.

HepG2 and LO2 were seeded in 24-well plates with a density of 5 × 10^4^ cells/well incubated for 12 h. Then two different groups (ICG-MPDA@SPIO/SA-PEI/AFP-Fth and ICG-MPDA@SPIO/PEI/AFP-Fth) were added into wells, after 1 h, 2 h and 4 h incubation, the cells were washed with PBS and fixed with 4% paraformaldehyde (PFA) for 15 min. DAPI was chose for nuclear staining. For the observation of the distribution of gene, FAM-labeled DNA (order of DNA, 5′-TCGGATACACCTA-3′, FAM-DNA purchased from Shanghai GeneChem Co., Ltd) was chosen as the alternative of AFP-Fth and MPDA@SPIO/SA-PEI/FAM-DNA was proceeded to the uptake assay for 1 h, 2 h and 4 h. The cellar Fe content was quantified by inductively coupled plasma mass spectrometry (ICP-MS, PerknElmer NexION300XX). In addition, we also dying lysosomal with Lyso-Tracker Red and co-localization with DNA.

### In vitro gene transfection

HepG2 cells were seeded in 24-well plate (5 × 10^4^ cell/well) and incubated with MPDA@SPIO/SA-PEI/AFP-GFP (5, 10, 20:1.25:1, w/w/w), MPDA@SPIO/ PEI/AFP-GFP (5, 10, 20:1.25:1, w/w/w), SA-PEI/AFP-GFP (1.25:1, w/w), PEI/AFP-GFP (1.25:1, w/w), and free AFP-GFP for 4 h respectively, the DNA content was fixed in 1.5 μg/well. Then the medium was removed and replaced with absolute medium followed by incubation for another 48 h. Finally, the green fluorescence protein (GFP) was observed by an inverted fluorescence microscope (OLYMPUS IX53, Japan). In the meantime, flow cytometry assay was also performed to quantitatively measurement of transfection efficiency. In brief, HepG2 cells were seeded in 6-well plates (1 × 10^5^ cells/well), the DNA content was fixed in 3 μg/well, MPDA@SPIO/SA-PEI/AFP-GFP (5, 10, 20:1.25:1, w/w/w), MPDA@SPIO/ PEI/AFP-GFP (5, 10, 20:1.25:1, w/w/w), SA-PEI/AFP-GFP (1.25:1, w/w), PEI/AFP-GFP (1.25:1, w/w), and free AFP-GFP were added, respectively. The rest procedures were repeated above. In the end, the cells were collected and washed by PBS for three times. The expression level of GFP was measured by the Flow Cytometer (Cytomics TM FC 500MCL, USA).

### Immunofluorescence distribution of E-selectin in HepG2 and LO2 cells

Immunofluorescence assay was performed to evaluate the difference in E-selectin expression between HepG2 and LO2. Briefly, HepG2 and LO2 were seeded in 24-well plates (5×10^4^ cells/well) that covered with coverslips and incubated overnight, then the cells were co-incubated with 5% FBS for 1 h at 25 °C for blocking the non-specific protein binding. Then cells were incubated with primary anti-E-selectin antibody (10 μg/mL) at 4 ℃ for overnight, then washed it by PBS for three time and followed by secondary antibody (FITC-labeled Goat Anti-Mouse IgG) stained for 10 min at room temperature. The fluorescence was detected and observed by CLSM.

### Analysis AFP-Fth transfection ability in HepG2 cells

To investigated the AFP expression difference between HepG2 and LO2 cells, proteins was extracted from HepG2 and LO2 cells with RIPA Lysis Buffer. Protein concentrations in cell lysates samples were measured using a BCA Protein Assay Kit (Thermo Fisher Scientific) according to the manufacturer’s protocol. Then, about 30 μg protein of each sample were electrophoresed on 12% SDS-PAGE, and transferred the gels to polyvinylidene difluoride (PVDF) membranes (Millipore, Inc., Billerica, MA) blocked with TBST containing 5% milk for 90 min. The membranes were incubated overnight with primary antibodies at 4 ℃ and then incubated with a horseradish peroxidase (HRP)-conjugated secondary anti-body for 90 min at room temperature. The bands’ signal was detected by ChemiDoc™ Touch Imaging System (Bio-Rad, Hercules, USA) with GAPDH as an internal reference.

After gene transfection of MPDA@SPIO/SA-PEI/AFP-Fth (20:1.25:1, w/w/w) MPDA@SPIO/PEI/AFP-Fth (20:1.25:1, w/w/w), SA-PEI/AFP-Fth (1.25:1, w/w), PEI/AFP-Fth (1.25:1, w/w) and free AFP-Fth, HepG2 cells were collected, and Western-blotting assay was performed to evaluate the expression of ferritin. All the procedures were repeated above.

To observe MR effect of nano-agent transfection cells, FAC (0.2 mM) was added to the culture medium after 24 h of transfection, and proceeded to incubate for 24 h. Then, discarded the medium and washed the plate for three times with PBS followed by dissociation with trypsin, and the obtained cells were mixed with 200μL agarose gel. All the samples were scanned by 3.0-T MRI Scanner (Philips, INGENIA 3.0T) in T2-weighted fast spin echo sequence (TR = 3000 ms, TE = 80 ms, matrix = 256 × 192, FOV = 120 mm × 90 mm, side thickness = 1.5 mm) to get T2 weighted images and the signal intensity value.

### In vivo MR imaging studies

To develop liver orthotopic xenograft mouse model, HepG2 cells were injected orthotopically into hepatic lobe of mouse at 5 × 10^4^ cells in 50 μL serum free DMEM medium. After 3 weeks post-tumor induction, HepG2 orthotopic tumor bearing mice were divided into three groups and administered with MPDA@SPIO/SA-PEI/AFP-Fth, MPDA@SPIO/PEI/AFP-Fth and SA-PEI/AFP-Fth by tail intravenous injection. Anesthesia mice treated with PBS were set as a control group. MRI was performed before and after administration of nanocomplexes at the time points of 6, 12, 24 and 48 h with a 3.0 MRI system (Philips, INGENIA) equipment of an animal coil for the mouse imaging. The parameters of transverse T2-weighted fast spin sequence were TR = 3000 ms, TE = 73.5 ms, FOV = 160 × 100 mm, Matrix 200 × 176, slice thickness = 1.5 mm. All the MR transverse images were stored and T2WI signal intensity of tumor area also recorded. To qualify the influence of contrast agents, the signal-to-noise ratio was adopted to evaluate through the signal intensity of the region of interest (ROI) that marked by red dotted circle. The relative signal-to-noise ratio (SNR = Smean (tumor signal)/NSD (standard deviation of the background signal)) was calculated to assess the T2 signal changes of tumor lesion. After MR examination, tumor specimens were harvested and used to analyze ferritin expression by Western blotting experiment. The GAPDH was set as a reference standard to normalize protein expression.

For histological analysis, tumor bearing liver specimens and major organs were fixed in 10% formalin and embedded in paraffin. Specimens were sectioned at 5 μm thickness and then stained with hematoxylin and eosin (H&E). For the immunohistochemical staining, Ki67 antibody was applied to the tumor sections.

## Supplementary Information

**Additional file 1:** **Fig. S1. **N2 adsorption–desorption isotherms of MPDA and MPDA@SPIO/SA-PEI/AFP-Fth. Fig. S2. (A) The hydrodynamic size of MPDA@SPIO/SA-PEI/AFP-Fth nanocomplexes over consecutive 7 days in water (a), PBS (b), DMEM (c), and PBS+10% FBS (inset is the photo of MPDA@SPIO/SA-PEI/AFP-Fth nanocomplexes dispersed in water (a), PBS (b), DMEM (c), and PBS+10% PBS (d) over a week), and (B) The TEM image of MPDA@SPIO/SA-PEI/AFP-Fth (w/w/w 20/1.25/1) in PBS+10% FBS after 7 days storage. Fig. S3. The Fe uptake in HepG2 and LO2 cells after treated with MPDA@SPIO/SA-PEI/AFP-Fth or MPDA@SPIO/PEI/AFP-Fth nanocomplexes for 1h, 2h and 4h. (n=3; **p<0.01)**.**

## Data Availability

All data generated or analyzed during this study are included in this article.
