# Supplementary material for: Sialic acid-engineered mesoporous polydopamine dual loaded with ferritin gene and SPIO for achieving endogenous and exogenous synergistic T2-weighted magnetic resonance imaging of HCC
Source: J Nanobiotechnology. 2021 Mar 17;19:76. doi: 10.1186/s12951-021-00821-8 (PMC7968241; doi:10.1186/s12951-021-00821-8)
Supplement: Supplementary file 1 — Additional file 1: Fig. S1. N2 adsorption–desorption isotherms of MPDA and MPDA@SPIO/SA-PEI/AFP-Fth. Fig. S2. (A) The hydrodynamic size of MPDA@SPIO/SA-PEI/AFP-Fth nanocomplexes over consecutive 7 days in water (a), PBS (b), DMEM (c), and PBS+10% FBS (inset is the photo of MPDA@SPIO/SA-PEI/AFP-Fth nanocomplexes dispersed in water (a), PBS (b), DMEM (c), and PBS+10% PBS (d) over a week), and (B) The TEM image of MPDA@SPIO/SA-PEI/AFP-Fth (w/w/w 20/1.25/1) in PBS+10% FBS after 7 days storage. Fig. S3. The Fe uptake in HepG2 and LO2 cells after treated with MPDA@SPIO/SA-PEI/AFP-Fth or MPDA@SPIO/PEI/AFP-Fth nanocomplexes for 1h, 2h and 4h. (n=3; **p<0.01). [file 12951_2021_821_MOESM1_ESM.docx]

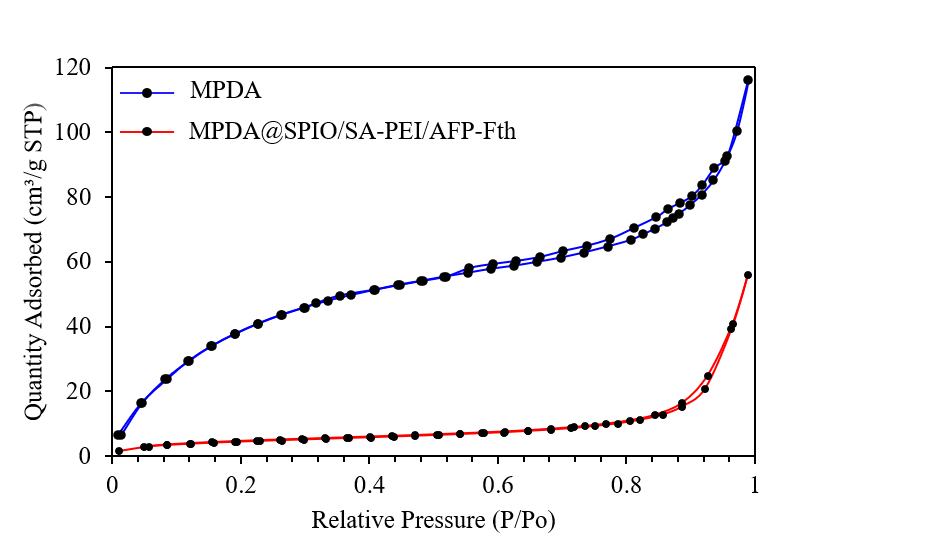


**Fig. S1.** N_2_ adsorption–desorption isotherms of MPDA and MPDA@SPIO/SA-PEI/AFP-Fth.


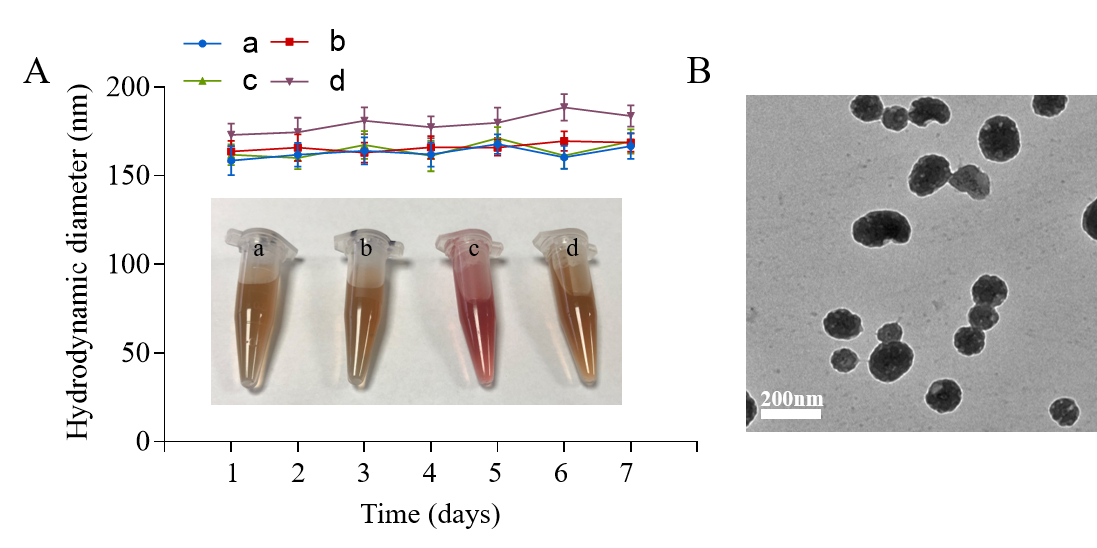


**Fig. S2.** (A) The hydrodynamic size of MPDA@SPIO/SA-PEI/AFP-Fth nanocomplexes over consecutive 7 days in water (a), PBS (b), DMEM (c), and PBS+10% FBS (inset is the photo of MPDA@SPIO/SA-PEI/AFP-Fth nanocomplexes dispersed in water (a), PBS (b), DMEM (c), and PBS+10% PBS (d) over a week), and (B) The TEM image of MPDA@SPIO/SA-PEI/AFP-Fth (w/w/w 20/1.25/1) in PBS+10% FBS after 7 days storage.


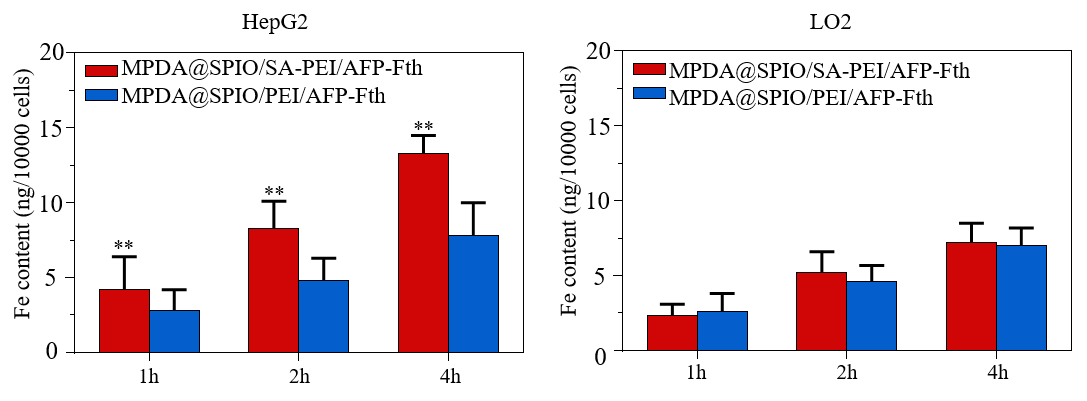


**Fig. S3.** The Fe uptake in HepG2 and LO2 cells after treated with MPDA@SPIO/SA-PEI/AFP-Fth or MPDA@SPIO/PEI/AFP-Fth nanocomplexes for 1h, 2h and 4h. (n=3; **p<0.01)
